# Effective Treatment of Folliculitis Decalvans Using Selected Antimicrobial Agents

**DOI:** 10.4103/0974-7753.66908

**Published:** 2010

**Authors:** Caulloo Sillani, Zhang Bin, Zhao Ying, Cai Zeming, Yang Jian, Zhang Xingqi

**Affiliations:** Department of Dermatology, The First Affiliated Hospital, Sun Yat-sen University, Guangzhou, Guangdong, People’s Republic of China

**Keywords:** Folliculitis decalvans, antimicrobial, primary cicatricial alopecia

## Abstract

Folliculitis Decalvans (FD) is a rare neutrophilic infammation of the scalp characterized by painful, recurrent purulent follicular exudation resulting in primary cicatricial alopecia. However, unclear etiology makes FD treatment a difficult task. A wide variety of topical and systemic agents have been tried previously, with varied results. We present here a case series report of a set of 13 patients with FD on antimicrobial therapy.

## INTRODUCTION

Folliculitis Decalvans (FD) is a rare neutrophilic inflammation of the scalp characterized by painful, recurrent purulent follicular exudation resulting in primary cicatricial alopecia. The superantigens theory,[1] with an abnormal host defense mechanism is widely accepted. However, unclear etiology makes FD treatment a difficult task. A wide variety of topical and systemic agents including antimicrobials, antifungals, retinoids, corticosteroids, as well as laser depilation treatment have been tried previously, with varied results.[2–7] We present here a case series report of a set of 13 patients with FD, who presented to our hair clinic for the past one year.

## MATERIALS AND METHODS

Thirteen Chinese patients presenting to our hair clinic between December 2008 and December 2009 with signs and symptoms of folliculitis Decalvans, were enrolled. A detailed clinical history was taken followed by a thorough scalp examination and routine systemic examination. The severity of the disease was assessed based on the clinical presentation, type of skin lesions, the area involved, and duration of the disease.

Pus swabs from intact pustules were taken for culture and antibiotic sensitivity test from six cases. Scalp biopsies were done on seven patients with signed consents. The protocol of antimicrobial therapy was based on the severity of the disease and antimicrobial sensitivity results. Patients with mild FD were started on Minocycline 100 mg twice daily, orally. Patients with moderate FD were given a combination of Minocycline 100 mg twice daily and Rifampicin 150 – 300 mg twice daily. The adjuvant drugs used were topical fusidic acid or mupirocin, selenium sulfide shampoo, oral compound glycyrrhizin, and zinc gluconate. The patients were reassessed after two weeks. Patients with deteriorating clinical symptoms, development of new skin lesions, and expansion of the involved area were given additional Clarythromycin 250 mg twice daily and/or Acitretin 10 mg once a day. The patients were assessed weekly or biweekly until satisfactory disease control, followed by monthly review.

## RESULTS

The results are listed in Table 1. There were 11 males (85%) and two females (15%) in the patient group, 15 to 66 years of age, with a mean age of 30.1±13.4 years. The course of the disease varied from two weeks to four years. The chief complaints were mainly painful scalp lesions accompanied by varying degrees of pruritis and hair loss. Androgenetic alopecia, acne, and atopic dermatitis were found in three, three, and one patient, respectively. No similar family history was found in any of the patients. 84.6% (11 cases) had vertex involvement. 61.5% (8 cases) had occipital involvement, among seven cases which overlapped with vertex involvement. The affected scalp area ranged from less than 5% to 20%. The skin lesions varied in morphology, they ranged from papular lesions with no exudation to pustular, boggy nodular masses with mild serous exudation on areas of hair loss ranging from 0.5 cm to < 2 cm in diameter. Tufted hairs were present in two patients. Eight cases were considered as mild and the rest were considered as moderate FD cases. Full blood count of the patients did not show any significant abnormal deviations. IgE was elevated in two patients (one of whom had a history of atopic dermatitis). Autoimmune antibody titers, HIV, and RPR / TPPA titers were negative in all the patients. Erythrocyte sedimentation rate (ESR) was not elevated in any of the patients. Seven cases who underwent histopathological examination were all confirmed as FD with typical folliculitis and features of scarring alopecia. *Staphylococcus aureus, Citrobacter koseri*, and coagulase-negative staphylococcus were grown in three cases, but no microorganisms were found in the other three cases. The duration of treatment varied from one month to more than one year. Minocycline 100 mg bid, given as monotherapy for an average of 5.7 weeks was able to clear the scalp lesions in seven patients; only one of them needed two weeks of oral Acitretin, and one of them had the disease relapse after eight months. A combination of Minocycline and Rifampicin for an average of 11.7 weeks was effective in treating three patients, in which Minocycline failed as a monotherapy. Combination of Clarythromycin and Rifampicin for an average of 10 weeks was also effective in clearing scalp lesions in two patients. Patient 11 [Figures 1–3] needed combinations of different antimicrobials with aggressive adjuvant therapy, as he neglected treatment in the beginning and his condition worsened significantly with scalp scarring. Only patient 11 had mild side effects from Rifampicin (nausea and vertigo); it was substituted by Clarythromycin after one month of therapy.

**Table 1:** Results

Patient no.	Age	Duration of disease / month	Severity of condition	Culture report (ND-Not Done)	Rx (wk-week/s; mo-month/s)	Duration of Rx	Follow-up
1	66	6	Mild	ND	Minocycline 100 mg bid × 2 wk	1 month	Relapse after 8 months
2	40	3	Mild	*Coagulase negative Staphylococcus*	Minocycline 100 mg bid × 4 wk	1 month	Good hair regrowth, no relapse
3	15	0.5	Moderate	ND	Minocycline 100 mg bid × 4 wk	1 month	Good hair regrowth, no relapse
4	23	1	Mild	ND	Minocycline 100mg bid × 2 wk	1 month	Good hair regrowth, no relapse
5	34	2	Mild	ND	Minocycline 100 mg bid × 8 wk	8 weeks	Good hair regrowth, no relapse
6	27	2	Mild	ND	Minocycline 100 mg bid × 8 wk	3 months	Good hair regrowth, no relapse
7	32	3	Mild	ND	Minocycline 100 mg bid × 3 mo Acitretin 10 mg OD × 2 wk	3 months	Good hair regrowth, no relapse
8	16	2	Mild	ND	Minocycline 100 mg bid × 2 wk Rifampicin 150 mg bid × 1 wk	1 month	Good hair regrowth, no relapse
9	25	2	Moderate	*Citrobacter koseri*	Minocycline 100 mg bid × 4 mo Rifampicin 300 mg bid × 3 mo	4 months	Good hair regrowth, no relapse
10	24	9	Moderate	Nil (twice)	Minocycline 100 mg bid × 4 mo Rifampicin 300 mg qd × 1 mo	4 months	Good hair regrowth, no relapse, on topical drugs
11	39	48	Moderate	*Staphylococcus aureus*	Minocycline 100 mg bid × 2 mo Clarythromycin 250 mg bid × 3 mo Acitretin 10 mg bid / qd × 1 mo Rifampicin 300 mg bid × 1 mo	> 1 year	Still under treatment, marked improvement, scalp skin scarring, loss of follicular ostia
12	30	1	Mild	Nil	Clarythromycin 250 mg bid × 2 wk Rifampicin 150 mg bid × 2 wk	1 month	Good hair regrowth, no relapse
13	20	36	Moderate	Nil	Clarythromycin 250 mg bid × 4mo Acitretin 10 mg qd-bid × 4 mo Rifampicin 300 mg qd × 3 mo	4 months	Partial hair growth, no relapse, on topical drugs

**Figure 1a:**
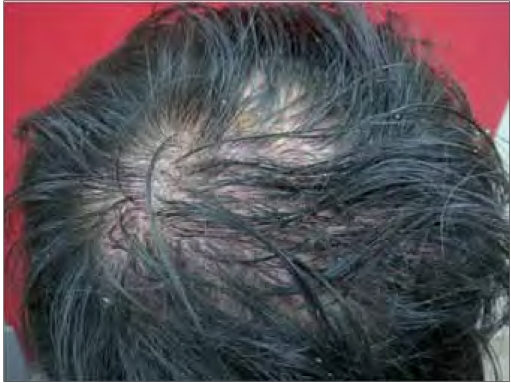
Patient 11 on presentation

**Figure 1b:**
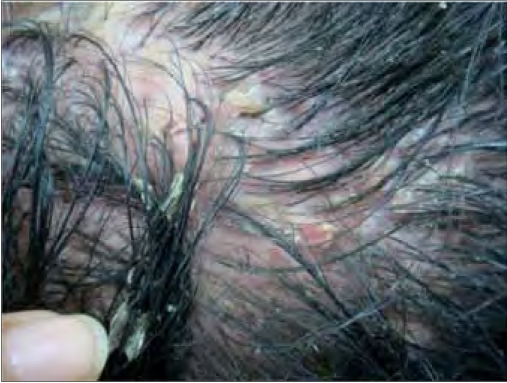
Patient 11 on presentation (close-up)

**Figure 2a:**
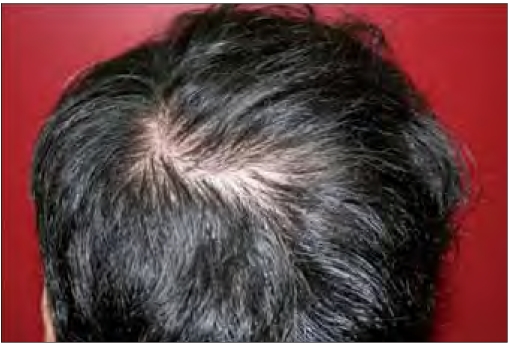
Patient 11 after five months of treatment

**Figure 2b:**
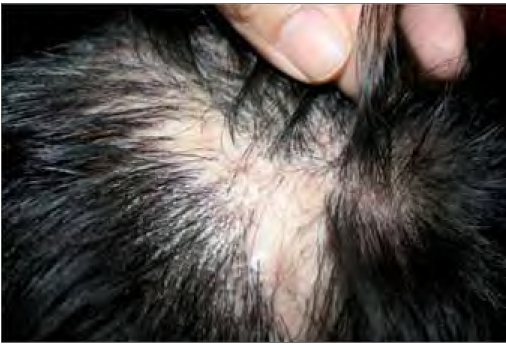
Patient 11 after five months of systemic antimicrobials (close-up) with scarring of scalp skin

**Figure 3a:**
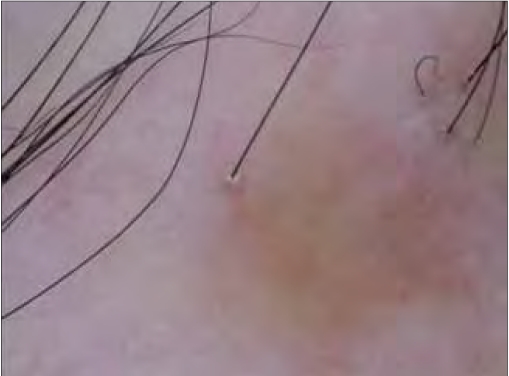
Patient 11 dermatoscopic changes – loss of follicular ostia

**Figure 3b:**
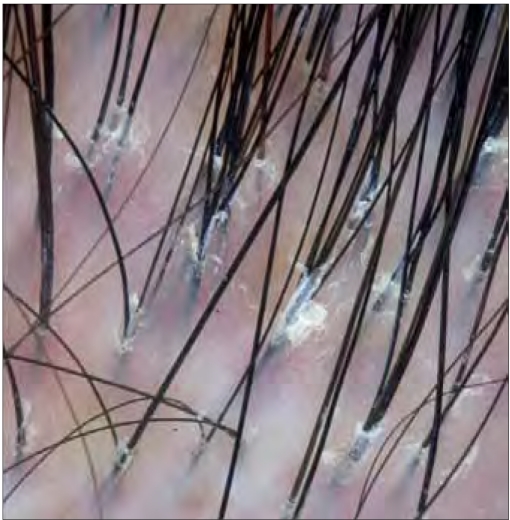
Patient 11 dermatoscopic changes – tufted hairs

## DISCUSSION

In our group of patients, 85% were males, which concurred with the male predominance in other studies as well (83%).[4] Among patients in whom bacterial and fungal cultures were carried out, only half of them yielded a positive result, and *Staphylococcus aureus* was not always the only pathogen found. However, these results could have been affected by contamination during the sampling procedures. Tufted hair was not a common finding in our patients, with an occurrence of 15% among all patients. Therefore, it could be a sign of severity of the disease and not an element for diagnosis.

Although non-antibacterials have also been used in the treatment of FD, the main aim has mainly been focused on the eradication of *S. aureus*. Rifampicin, which acts by inhibiting DNA-dependent RNA polymerase activity in susceptible cells, is very effective against *S. aureus*. Minocycline hydrochloride, which is a semi-synthetic derivative of tetracycline, has a bacteriostatic effect, as it inhibits protein synthesis of Gram negative and Gram positive microorganisms, including *S. aureus*. Some studies have already proved the effectiveness of Rifampicin,[4,8,9] however, due to rapid emergence of antibiotic resistance, it has been used in combination with other antimicrobials. The use of Clarythromycin, a macrolide antimicrobial, which binds to the 50S ribosomal subunit of susceptible microorganisms and inhibits protein synthesis, has also been suggested earlier.[1] We thus preferred to use Minocycline as the drug of first choice, while Rifampicin was used additionally when Minocycline monotherapy was ineffective. In one of our patients, Rifampicin was substituted by Clarythromycin due to mild side-effects; Clarythromycin proved to be as effective.

The antimicrobials used in this study are only a few of the large variety available for successful FD treatment, however, each drug needs proper tailoring according to the patient’s condition, along with clinical assessment and laboratory investigations. Cosmetically acceptable results have been attained in most of the patients in a relatively short time. The antimicrobials used are also safe and their side-effects when used in separate short courses are mild. Most of our patients (9/13) are still disease-free with good hair regrowth, while the others are being controlled either by topical (2/13) or systemic antibiotics (1/13). Thus, early and effective treatment of FD is needed to prevent progression of the disease and scarring of the scalp skin.

## CONCLUSION

Minocycline is a very effective antimicrobial agent in mild cases, but administration of Rifampicin or Clarythromycin in combination with Minocycline is helpful in moderate or resistant cases. An early treatment of FD, with proper antimicrobials, is important for preventing total destruction of hair follicles leading to scarring alopecia.
